# Retraction: Rehmannia Glutinosa Extract Activates Endothelial Progenitor Cells in a Rat Model of Myocardial Infarction through a SDF-1 α/CXCR4 Cascade

**DOI:** 10.1371/journal.pone.0217149

**Published:** 2019-05-20

**Authors:** The PLOS ONE Editors

Following publication of this article [1], concerns were raised about possible duplication of images within Figures 2, 5, 6 and 7.

Specifically,

- In Figure 2A there is a region of partial overlap between the NS-b and RGE-b panels.
- In Figure 5A the VEGFR2 NS-b panel and the VEGFR2 RGE-b panel appear similar; there is a region of partial overlap between the CD133 NS-b panel and the CD133 RGE-b panel.
- In Figure 6A there is a region of partial overlap between the NS-b and RGE-b panels.
- In Figure 6C the β-actin western blot bands for NS-m, NS-MI, and RGE-MI have a similar appearance. The CXCR4 and SDF-1 bands for RGE-MI have a similar appearance.
- In Figure 7, the actin bands for 10 and 25 μg/ml in 7B have a similar appearance; the CXCR4 band for 25 μg/ml in 7B has a similar appearance to the actin band labelled C in 7C; the actin band for AMD in 7B has a similar appearance to the CXCR4 band for 72h in 7C; in 7C, the actin band for 6h and the CXCR4 band for 12h have a similar appearance; and the actin and CXCR4 bands for 24h have a similar appearance in 7C.
- In Figure 7D, the two left-hand histograms are duplicates.

The authors have stated that errors were introduced at the stage of revising the manuscript. In follow up to the concerns raised, the authors provided the uncropped images underlying the above panels. They also provided replacement immunohistochemistry images and replicate uncropped immunohistochemistry images from all experimental animals to address the concerns raised regarding Figures 2, 5, and 6, a replacement histogram for Figure 7D, and replacement western blots for Figures 6 and 7.

The *PLOS ONE* Editors evaluated the information and data provided and consulted with a member of the Editorial Board. Partial overlap in immunohistochemistry images was noted between tissue samples derived from different treatment groups, according to figure labels, and this concern could not be resolved by examination of the uncropped image files. Additionally, the evaluation suggested that incorrect western blot bands were used in the published figures for Figure 6C β-actin bands and the SDF-1α RGE-MI band; Figure 7B the actin band for 10 μg/ml; and Figure 7C the actin bands for 6h, 12h, and 24h, and the CXCR4 bands for C, 12h, 24h, and 72h. Regarding the western blot methodology, the authors clarified that protein expression was normalized to actin levels; however, actin samples were run on different blots. For Figures 6 and 7, individual representative bands were taken from several different blots in the original published figures. The authors provided alternative continuous blots in support of their findings (in which all bands for the figure were present on adjacent lanes in the same blot, with a corresponding separate continuous blot for the actin controls). These alternative blots were carried out during the original study and form part of the underlying data for the quantifications reported in the charts in Figures 6D and 7D-E.

The *PLOS ONE* Editors acknowledge that the authors identified inaccuracies in some of the images listed and requested corrections for those both prior to and shortly after publication of the article, and we regret that these concerns were not addressed earlier. In light of concerns about the reliability of the reported data, figure preparation, and conclusions, the *PLOS ONE* Editors retract this article.

Y-BW, Y-FL, X-TL, F-FY, BW, W-WB, Y-XZ did not agree with retraction.

## References

[1] Wang Y-B, Liu Y-F, Lu X-T, Yan F-F, Wang B, Bai W-W, et al Rehmannia Glutinosa Extract Activates Endothelial Progenitor Cells in a Rat Model of Myocardial Infarction through a SDF-1 α/CXCR4 Cascade. PLoS ONE. 2013;8(1): e54303 10.1371/journal.pone.0054303 23349848 PMC3548813

